# Defining the Skeletal Myogenic Lineage in Human Pluripotent Stem Cell-Derived Teratomas

**DOI:** 10.3390/cells11091589

**Published:** 2022-05-09

**Authors:** Matthew P. Pappas, Ning Xie, Jacqueline S. Penaloza, Sunny S. K. Chan

**Affiliations:** 1 Department of Pediatrics, University of Minnesota, Minneapolis, MN 55455, USA; pappa067@umn.edu (M.P.P.); xiening0612@gmail.com (N.X.); penal007@umn.edu (J.S.P.); 2 Stem Cell Institute, University of Minnesota, Minneapolis, MN 55455, USA; 3 Lillehei Heart Institute, University of Minnesota, Minneapolis, MN 55455, USA

**Keywords:** pluripotent stem cells, myogenic development, muscle stem cells, satellite cells

## Abstract

Skeletal muscle stem cells are essential to muscle homeostasis and regeneration after injury, and have emerged as a promising cell source for treating skeletal disorders. An attractive approach to obtain these cells utilizes differentiation of pluripotent stem cells (PSCs). We recently reported that teratomas derived from mouse PSCs are a rich source of skeletal muscle stem cells. Here, we showed that teratoma formation is also capable of producing skeletal myogenic progenitors from human PSCs. Using single-cell transcriptomics, we discovered several distinct skeletal myogenic subpopulations that represent progressive developmental stages of the skeletal myogenic lineage and recapitulate human embryonic skeletal myogenesis. We further discovered that ERBB3 and CD82 are effective surface markers for prospective isolation of the skeletal myogenic lineage in human PSC-derived teratomas. Therefore, teratoma formation provides an accessible model for obtaining human skeletal myogenic progenitors from PSCs.

## 1. Introduction

Skeletal muscle homeostasis is primarily maintained by endogenous PAX7^+^ skeletal muscle stem cells (a.k.a. satellite cells) [1,2,3,4,5]. Upon injuries, skeletal muscle stem cells become activated, proliferate, and differentiate into myoblasts, which subsequently fuse into myofibers. The potency of these cells to repair damaged muscles makes them an excellent cell source for regenerative medicine. However, they are rare, making up only 1–2% of the mononuclear fraction of muscle tissue [6], and their clinical use is thus limited.

Differentiation of pluripotent stem cells (PSCs), including embryonic stem cells (ESCs) and induced PSCs (iPSCs), is a promising method of obtaining large quantities of skeletal myogenic progenitors, because PSCs are readily available and expandable [7,8,9,10,11]. In recent years, several protocols were developed to differentiate PSCs into the skeletal myogenic lineage [12,13,14,15,16,17,18,19]. Nevertheless, whereas the skeletal myogenic cells thus derived were able to form multinucleated myotubes in vitro, their regenerative potential in vivo was somewhat limited—only a few hundred fibers were observed even though millions of cells were transplanted [13,19,20]. In contrast, we have recently shown that skeletal myogenic progenitors obtained from mouse PSC-derived teratomas have an exceptional regenerative potency similar to endogenous adult muscle stem cells [21]. A mere 40,000 mouse teratoma-derived skeletal myogenic progenitors are capable of regenerating ~80% of total muscle volume. Furthermore, these skeletal myogenic progenitors are expandable in vitro for more than a month while maintaining high engraftment potential [22]. We sought to determine whether a similar in vivo differentiation strategy is applicable to human PSC-derived teratomas. Here, we characterized the development of skeletal myogenic progenitors in human PSC-derived teratomas and identified surface markers for their prospective isolation.

## 2. Experimental Procedures

### 2.1. Animals

Animal housing, husbandry, and experimental procedures involving animals, including teratoma formation and cell transplantation described in this study, were carried out according to protocols (no. 1903-36840A) approved by the University of Minnesota Institutional Animal Care and Use Committee and under institutional assurances of AAALAC accreditation (no. 000552, as of November 2015), USDA research facility registration (USDA No. 41-R-0005), and PHS Animal Welfare Assurance approval (A3456-01). The NSG-mdx^4Cv^ mice were generated by crossing NOD.Cg-Prkdc^scid^Il2rg^tm1Wjl^/SzJ (NSG) mice and B6Ros.Cg-Dmd^mdx-4Cv^/J (mdx^4Cv^) mice [23] and maintained in autoclaved cages. NSG-mdx^4Cv^ mice of both sexes (3–4 months old) were used for experiments.

### 2.2. Pluripotent Stem Cell Culture

H1 human ESCs were obtained from WiCell (WA01, Madison, WI, USA). EGFP was engineered into the AAVS1 safe harbor locus of H1 ESCs using zinc finger nucleases as described previously [24]. The 6B4 human iPSCs (derived from adult conjunctival cells) [25] and PCBC16iPS human iPSCs (derived from neonatal dermal fibroblasts) [26] were gifts from Dr. James Dutton at the University of Minnesota (Minneapolis, MN, USA). PSCs were maintained in TeSR-E8 (Stem Cell Technologies no. 05990, Vancouver, BC, Canada) on Cultrex-coated dishes (R&D Systems no. 3434-010-02, Minneapolis, MN, USA) at 37 °C. Cells were passaged at 50% confluency with Accutase (Innovative Cell Technologies no. AT-104, San Diego, CA, USA).

### 2.3. Teratoma Formation

Human ESCs or iPSCs were injected into tibialis anterior (TA) muscles of immunodeficient NSG-mdx4Cv mice to generate teratomas. Recipients were anesthetized with ketamine (150 mg/kg, i.p., Akorn no. NDC:59399-114-10, Lake Forest, IL, USA) and xylazine (10 mg/kg, i.p., Akorn no. NDC:59399-111-50). For each recipient mouse, 1 million undifferentiated ESCs or iPSCs in TeSR-E8 mixed with Cultrex at 1:1 (total 20 μL) were injected into the left TA muscle using a Hamilton syringe (Hamilton, Reno, NV, USA). Only 1 teratoma was produced for each mouse. Mice were daily monitored after cell injection.

### 2.4. Teratoma Harvest

Teratomas at 3–8 weeks were harvested and cut into ~2-mm pieces. Teratoma pieces were then put into a digestion buffer containing Dulbecco’s Minimum Essential Medium/High Glucose (HyClone no. SH30243.01, Logan, UT, USA), 2 mg/mL Collagenase II (Gibco no. 17101-015, Gaithersburg, MD, USA), and 1% penicillin/streptomycin (P/S) (Life Technologies no. 15140-122, Grand Island, NY, USA) on a shaker at 250 rpm, 37 °C for 30 min. Digested cell slurry was then filtered through 40 μm cell strainers and spun down, and the filtered cell pellet was washed with rinsing buffer consisting of Ham’s/F-10 medium (Caisson Labs no. HFL01, Smithfield, UT, USA), 10% horse serum (HyClone no. SH30074.03), 1% HEPES buffer solution (Caisson Labs no. HOL06), and 1% P/S and kept on ice (first-pass). Undigested tissues were further incubated in the digestion buffer on the shaker for another 30 min, then filtered through 40 μm cell strainers, spun down, and the cell pellet was washed with rinsing buffer (second-pass). Cells from first-pass and second-pass were combined, resuspended in FACS buffer (PBS (HyClone no. SH30256.01) with 0.2% FBS (Gemini Bio-Products no. 100-119, West Sacramento, CA, USA)) and kept on ice until downstream processing. An 8-week-old teratoma yielded approximately 200 million mononuclear cells (see Section 3 below).

### 2.5. Fluorescence Activated Cell Sorting (FACS)

Dissociated cells were incubated with fluorophore-conjugated antibodies on ice for 30 min. Stained cells were washed twice in FACS buffer and resuspended in FACS buffer with propidium iodide (PI, 1 μg/mL, Sigma no. P4170, St. Louis, MO, USA) for FACS analysis. PI was used as a live/dead cell indicator and only live cells (PI–) were counted. Cells were analyzed and sorted by BD FACSAriaII (BD Biosciences, San Diego, CA, USA) with FACSDiva software (BD Biosciences). Sorted cells were collected into cell culture medium and kept on ice until downstream processing. FACS plots were generated using FlowJo (FLOWJO LLC, Ashland, OR, USA). Antibodies used were APC anti-CD56 (Thermo Fisher Scientific 17-0567-42, RRID:AB_10597454, Waltham, MA, USA) APC anti-CD82 (R&D Systems no. FAB4616A, RRID:AB_2076404) and PE anti-ERBB3 (BioLegend no. 304706, RRID:AB_2099569, San Diego, CA, USA), all at 0.5 μL per million cells.

### 2.6. Single-Cell RNA-Seq Preprocessing and Quality Control

Eight-week EGFP-labeled H1 ESC-derived teratoma cells were sorted for GFP by FACS and were then loaded into a 10× Genomics Chromium system (Pleasanton, CA, USA) for single-cell capture. RNA-seq was performed using an Illumina HiSeq2500 (San Diego, CA, USA). Raw data were then pre-processed with Cell Ranger (v3.0.2) using the hg38 human genome as provided by 10× Genomics. The single-cell RNA seq dataset was analyzed using the R package Seurat [27,28,29]. Cells with fewer than 200 unique genes detected were filtered out to remove low-quality droplets (Figure S2A). Cells with more than 4000 unique genes detected were removed to prevent the inclusion of doublets (Figure S1A). Cells within which greater than 5% of reads were of mitochondrial origin were filtered out to remove dead or low-quality cells (Figure S2B). Expression levels were normalized using default parameters in Seurat [27,28,29]. The 2000 genes with the highest standardized variance in expression level across cells were selected for use in subsequent analysis (Figure S2C).

### 2.7. Principal Component Analysis

To prevent highly expressed genes from dominating principal component analysis expression levels for each gene were scaled such that the mean expression level was equal to 0 and the variance was equal to 1. Principal component analysis was conducted on the population of cells to determine dimensions of correlated variance in gene expression. The first 19 principal components characterized the dimensionality of variation in population gene expression and were selected for use in subsequent analyses (Figure S2D).

### 2.8. Cell Clustering and Dimension Reduction

K-nearest-neighbor (KNN) clustering was performed in Seurat [27,28,29] with an optimized resolution of 0.95 and default parameters. Resolution was optimized by Cluster tree analysis (Figure S2E) using the R package Clustree [30]. Uniform manifold approximation and projection (UMAP) dimension reduction was performed to display cell clusters in a two-dimensional space [31].

### 2.9. Trajectory Analysis

Filtered cells were used for independent principal component analysis using default parameters in Monocle3 [32,33,34,35]. UMAP dimension reduction was performed on the first 25 principal components. Trajectory learning was then applied to the myogenic subset of cells from the bulk population using default parameters.

### 2.10. GO Term Enrichment Analysis

Starting with a list of genes differentially expressed in a subset of cells, an expression score of each gene of interest was calculated. The score was generated by determining the ratio of cells within the subset expressing each gene as compared to the remainder of the bulk population. Then the differential gene list was ranked by expression score and GO biological process enrichment analysis was performed using Gorilla [36,37]. For comparing the list of differentially expressed genes for a given subcluster to other closely related subclusters (after exclusion of unrelated cells from the bulk population), PANTHER was used for the GO biological process enrichment analysis [38].

### 2.11. Integration of Single-Cell Datasets

Sequencing data were integrated using the standard Seurat v4 integration workflow with 2000 variable genes and 22 PCs [39].

### 2.12. Diffusion Map Generation

Diffusion map construction used detected genes of the top 2000 variable genes from the human skeletal myogenic progenitor tissue samples from Xi et al. [40] and the DiffusionMap function of Destiny [41] using standard parameters.

### 2.13. Cluster Expression Correlation Analysis

Average expression was calculated for the top 2000 variable genes from the human skeletal myogenic progenitor tissue samples from Xi et al. [40] in each integrated cluster of interest using the Seurat AverageExpression function [39]. Pearson’s correlation analysis was subsequently performed pairwise across clusters.

### 2.14. Quantitative PCR

Total RNA was extracted using RNeasy Mini Kit (QIAGEN no. 74106, Valencia, CA, USA), and subsequent genomic DNA removal and reverse transcription were performed using Verso cDNA Synthesis Kit (Thermo Scientific no. AB1453A, Pittsburgh, PA, USA). Quantitative polymerase chain reaction (qPCR) was performed in triplicates using TaqMan probes (Applied Biosystems, Carlsbad, CA, USA) and Premix Ex Taq (probe qPCR) master mix (Clontech no. RR39WR, Mountain View, CA, USA). Expression of individual genes was subsequently analyzed by the ΔCt method in relative to the expression of the housekeeping gene *Gapdh* in a QuantStudio 6 Flex Real-Time PCR System using QuantStudio Real-Time PCR Software (both Applied Biosystems). Taqman probes used were: *PAX7* Hs00242962_m1, *MYOD1* Hs00159528_m1, and *GAPDH* Hs99999905_m1.

### 2.15. Immunoblotting

Protein extracts were separated by electrophoresis on 10% SDS-polyacrylamide gels and then transferred to PVDF membranes. Membranes were blocked with 5% nonfat dry milk in Tris-buffered saline and 0.1% Tween 20 (Bio-Rad no. 170-6531, Hercules, CA, USA) for 1 h, and then incubated with the primary antibody overnight at 4 °C, followed by incubation with secondary antibody peroxidase-conjugated anti-mouse IgG (1:10,000, GE Healthcare no. NA931, RRID:AB_772210, Chicago, IL, USA) for 1 h. Detections were carried out using a chemiluminescence detection substrate (Thermo Fisher Scientific no. 32106). Primary antibodies used were mouse anti-PAX7 at 1:10, Developmental Studies Hybridoma Bank (DSHB) no. PAX7, RRID:AB_528428, Iowa City, IA, USA; mouse anti-MYOD1 at 1:1000, BD Biosciences no. 554130, RRID:AB_395255, Franklin Lakes, NJ, USA; and HRP-conjugated GAPDH at 1:10,000, Proteintech no. HRP-60004, RRID:AB_2737588, Rosemont, IL, USA.

### 2.16. Skeletal Myogenic Cell Culture

Human teratoma-derived skeletal myogenic cells were maintained in SkGM-2 (Lonza no. CC-3245, Basel, Switzerland) on 0.1% gelatin-coated dishes (Sigma no. G9391, St. Louis, MO, USA). For differentiation, cells were cultured in N2 medium: DMEM/F12, 1% ITS-A (Gibco no. 51300-044), 1% N2 (Gibco no. 17502-048), 1% Glutamax (Life Technologies no. SCR006), 1% penicillin/streptomycin (Life Technologies no. 15140-122) supplemented with 10 μM SB-431542 (APExBIO no. A8249, Houston, TX, USA) for 4 days.

### 2.17. Immunostaining

Cell cultures were fixed with 4% paraformaldehyde (PFA) (Sigma no. P6148) for 20 min, rinsed three times in PBS, then permeabilized with 0.3% Triton X-100 (Sigma no. X100) for 30 min, followed by blocking with 3% bovine serum albumin (BSA) (Thermo Fisher Scientific no. BP1605-100) for 1 h, all at room temperature. Primary antibodies were diluted in 3% BSA and incubated overnight at 4 °C. Cultures were then washed three times in PBS and incubated with appropriate coupled secondary antibodies in 3% BSA for 1 h at room temperature. Cultures were counterstained with DAPI for 15 min and washed 3 times in PBS before analysis. Primary antibody used was mouse anti-MHC at 1:20, DSHB no. MF-20, RRID:AB_2147781, and secondary antibody used was goat anti-mouse Alexa Fluor 555 (Life Technologies). Fluorescent images were captured with a Zeiss AxioObserver Z1 inverted microscope with an AxioCamMR3 camera using the ZEN software (Zeiss, Jena, Germany). Image analysis was performed in ImageJ using binary-transformed area quantification of MHC+ fiber area.

### 2.18. Statistics Analysis

Data are presented as mean ± SEM. Student’s *t*-tests were used for comparisons between two groups. One-way ANOVA with Tukey’s post-hoc tests were used for comparisons among three or more groups. Differences are considered to be statistically significant at the *p* < 0.05 level.

## 3. Results

### 3.1. Human H1 ESC-Derived Teratomas Contain Skeletal Myogenic Progenitors

We previously reported that mouse PSC-derived teratomas contained skeletal muscle progenitors with exceptional regenerative capacity [21,22]. We wished to evaluate whether the teratoma formation approach is also capable of producing skeletal muscle progenitors from human PSCs. We started with EGFP-labeled H1 human ESCs, a commonly available and widely studied ESC line [42]. To generate teratomas, we implanted H1 ESCs in the tibialis anterior (TA) muscles of NSG-mdx^4Cv^ mice [23]. We chose the TA muscles because our preliminary work showed that skeletal myogenic cells were more readily found in teratomas formed in the TA muscle than in the flank (data not shown). We harvested teratomas 3–8 weeks later and assessed the development of the skeletal myogenic lineage (Figure S1A–G). Teratomas grew slowly at the beginning, began to grow more rapidly starting at week 5, and by week 8 reached on average of 2 cm in length and yielded approximately 200 million mononuclear cells per teratoma. The muscle stem cell factor *PAX7* and the muscle specification factor *MYOD1* were detected as early as weeks 3 and 7, respectively, and by week 8 they were readily observed at the protein level. In vitro cultures of total teratoma cells also revealed the emergence of myosin heavy chain (MHC)^+^ myotubes upon terminal differentiation at week 5 and these myotubes became more prominent by week 8. We decided to use 8-week-old teratomas at their skeletal myogenic prime for subsequent experiments.

**Figure 1:**
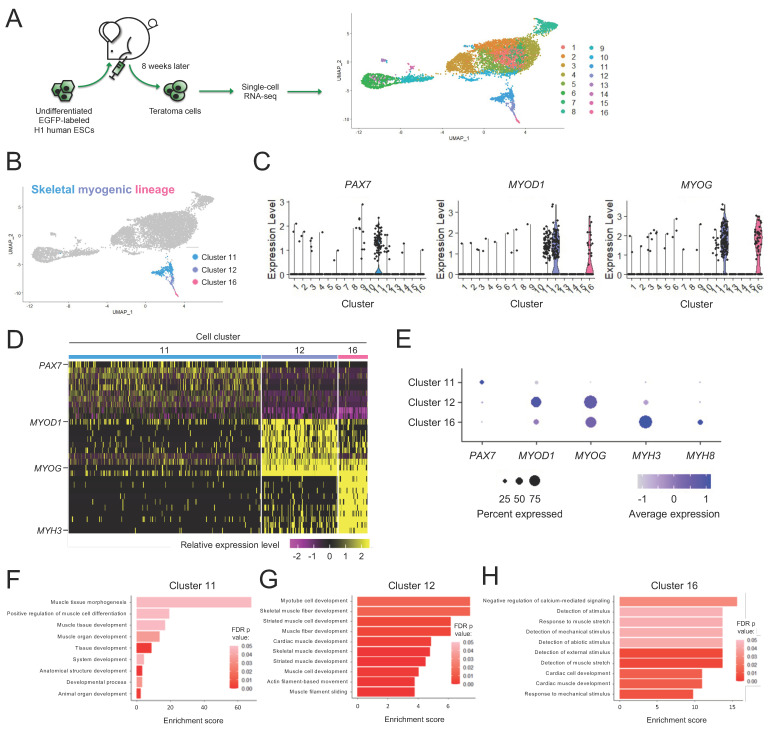
Skeletal myogenic cells are found in human H1 ESC-derived teratomas. (**A**) Schematic illustrating single-cell RNA-seq analysis of human H1 ESC-derived teratoma cells. (**B**) Cells with expression profiles resembling the skeletal myogenic lineage are found in Clusters 11, 12, and 16. (**C**) Violin plots of skeletal myogenic regulatory factors *PAX7, MYOD1* and *MYOG* showing that they primarily express in Clusters 11, 12, and 16. (**D**) Heatmap showing within the three skeletal myogenic clusters the top 10 enriched genes, among them several key myogenic factors. (**E**) Dot plot showing the enrichment of several skeletal myogenic regulatory factors in Clusters 11, 12, and 16. (**F**–**H**) Gene Ontology biological processes that are enriched are shown for Cluster 11 (**F**), Cluster 12 (**G**), and Cluster 16 (**H**). Significance was determined using false discovery rate-corrected (FDR) *p* values at < 0.05.

Despite recent efforts, there currently remains little consensus on how to identify and isolate the skeletal myogenic lineage derived from human PSCs [20,40,43]. To define the skeletal myogenic lineage for its prospective isolation in human teratomas, we performed single-cell RNA-seq. From 6895 H1 ESC-derived teratoma cells, we discovered 16 clusters with distinct gene expression profiles (Figure 1A and Figure S2). We reasoned that the skeletal myogenic lineage in teratomas might be composed of cells at various developmental stages, spanning PAX7^+^ progenitors to MYOD1^+^ and MYOG^+^ myoblasts. We subsequently found that these skeletal myogenic factors were enriched in Clusters 11, 12, and 16 (Figure 1B–D). We determined that these clusters were the skeletal myogenic subset of human ESC-derived teratoma cells.

### 3.2. Subpopulations of the Skeletal Myogenic Lineage Are Distinguished by Developmental Stage

We next wished to further define the expression profiles of these skeletal myogenic clusters. We found that these clusters expressed stage-specific markers of muscle development in a progressive manner, with muscle stem cell factor *PAX7* highly expressed in Cluster 11, skeletal myogenic specification/determination factors *MYOD1* and *MYOG* in Clusters 12 and 16, and myogenic structural components *MYH3* and *MYH8* in Cluster 16 (Figure 1E). Gene ontology (GO) enrichment analysis of differentially expressed genes further showed enrichment of biological processes related to early development of muscle tissues in Cluster 11, muscle differentiation and functional development processes in Cluster 12 and operation of skeletal muscle machinery in Cluster 16 (Figure 1F–H). These results indicated that Clusters 11, 12, and 16 were skeletal myogenic populations at various stages of development.

**Figure 2:**
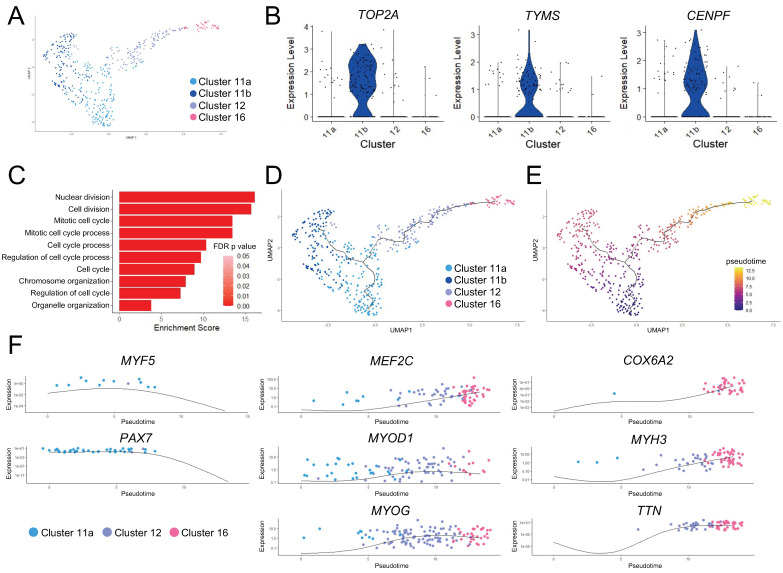
Pseudotime ordering and trajectory analysis of the skeletal myogenic lineage. (**A**) UMAP of the three skeletal myogenic clusters. Cluster 11 is subdivided into Subclusters 11a and 11b (see main text for details). (**B**) Violin plots showing that cell cycle-related genes *TOP2A*, *TYMS*, and *CENPF* are enriched in Subcluster 11b. (**C**) GO term enrichment in Subcluster 11b. (**D**,**E**) Pseudotime ordering and trajectory analysis of (**A**). The line indicates the differentiation trajectory. (**D**) Cell cluster identities are shown along with (**E**) values of pseudotime. Higher values in pseudotime indicate cells further down the differentiation trajectory. (**F**) Expression of key skeletal myogenic genes is shown over pseudotime along the skeletal myogenic differentiation trajectory.

We wished to further establish the relationship among the three skeletal myogenic clusters. We first performed independent k-means clustering on these skeletal myogenic clusters and found two subclusters in Cluster 11: Subclusters 11a and 11b (Figure 2A). Gene expression and GO enrichment analysis suggested that Subcluster 11b cells were cycling and proceeding through mitosis (Figure 2B,C).

We next performed pseudotime ordering and trajectory analysis to characterize the change in gene expression states that might drive the progression in functional states of skeletal myogenic cells. This analysis established a branched trajectory over pseudotime, with Subcluster 11a cells proceeding toward Subcluster 11b cell state or through Cluster 12 toward Cluster 16 cell state (Figure 2D,E). Together with the previously discussed gene expression differences between these clusters, we classified Cluster 11 cells as skeletal myogenic progenitors, with Subcluster 11b cells being those actively undergoing cell cycling or self-renewal. The other branch of the trajectory of Cluster 11 cells represented skeletal myogenic progenitor cells undergoing further differentiation, with Cluster 12 cells in an early myoblast-like state, and Cluster 16 cells in a late myoblast-like state.

This differentiation trajectory was further characterized by changes in key skeletal myogenic factors (Figure 2F). We found downregulation of early skeletal myogenic transcription factors *PAX7* and *MYF5* over pseudotime. The downregulation of these early factors was paired with contemporaneous upregulation of factors that drive skeletal myogenic differentiation such as *MYOD1*, *MEF2C*, and *MYOG*. The cells furthest along the differentiation trajectory began to express the striated muscle-specific cytochrome c oxidase subunit 6 gene (*COX6A2*), along with genes encoding muscle function proteins, such as titin (*TTN*) and embryonic myosin heavy chain (*MYH3*). Taken together, these data indicated that skeletal myogenic differentiation was actively occurring in human ESC-derived teratomas.

### 3.3. Skeletal Myogenic Differentiation in ESC-Derived Teratomas Recapitulates Human Embryonic Skeletal Myogenesis

**Figure 3:**
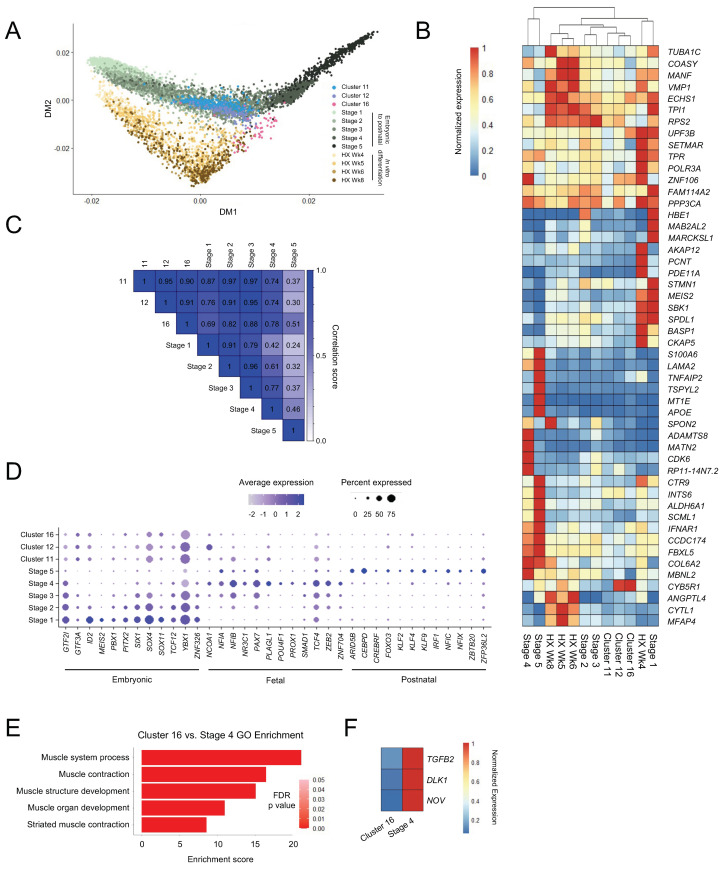
Skeletal myogenesis in teratomas recapitulates muscle development in vivo. (**A**) Diffusion map of teratoma-derived skeletal myogenic progenitors from the current study (Clusters 11, 12, and 16), and human muscle tissues (Stage 1–5) and in vitro differentiated cells (HX Wk4–8) from Xi et al. [40]. Human samples were collected from embryonic week 5 limb muscles to adult muscle biopsies, and in vitro cells were collected at 4–8 weeks of skeletal myogenic differentiation [40]. (**B**) Heatmap showing normalized expression of the top 50 differentially expressed genes among the skeletal myogenic populations. (**C**) Transcriptome correlation analysis of teratoma-derived and human skeletal myogenic cells. (**D**) Dot plot showing expression profiles of various transcription factors across embryonic, fetal, and postnatal stages for the skeletal myogenic populations. (**E**) Top five GO terms of differentially expressed genes that distinguish Cluster 16 cells from Stage 4 cells. (**F**) Differentially expressed genes in the TGFβ (*TGFB2)* and Notch (*DLK1, NOV*) signaling pathways between Cluster 16 cells and Stage 4 cells.

While methods exist to derive PAX7^+^ skeletal myogenic progenitors from PSCs, differentiation approaches capable of producing cell types that are functionally equivalent to postnatal skeletal muscle stem cells remain coveted. Having characterized skeletal myogenic progenitors in human ESC-derived teratomas, we next wished to compare these cells to skeletal myogenic progenitors from other sources. We integrated the present study with single-cell gene expression data [40] of skeletal myogenic progenitor populations from human embryonic/fetal and postnatal muscle tissues and from ESC-derived skeletal myogenic progenitors differentiated in vitro [44]. These cells were bioinformatically categorized into five developmental stages: Stage 1 (corresponding to mostly week 5–6), Stage 2 (mostly week 6.5–7.25), Stage 3 (mostly week 7.75–9), Stage 4 (mostly week 12–18), and Stage 5 (mostly juvenile and adult) [40]. From this skeletal myogenic developmental continuum, we constructed a diffusion map [41] to evaluate the degree to which skeletal myogenic development in ESC-derived teratomas resembled in vitro ESC skeletal myogenic differentiation and human embryonic-to-postnatal skeletal myogenesis. Remarkably, we found that the distribution of teratoma-derived skeletal myogenic cells (Clusters 11, 12 and 16) overlapped closely with those developed during human skeletal myogenesis (Stages 1–5) (Figure 3A). In contrast, the development of teratoma-derived skeletal myogenic cells diverged from in vitro ESC skeletal myogenic differentiation (HX Wk4–8), which appeared to form its own distinct development trajectory (Figure 3A). Independent hierarchical clustering using highly variably expressed genes also suggested that Stage 2/3 embryonic/fetal skeletal myogenic cells were more closely related to teratoma-derived skeletal myogenic cells (Clusters 11, 12, and 16) than to in vitro differentiated cells (HX Wk4–8) (Figure 3B).

The similarity between teratoma-derived and human skeletal myogenic cells were further demonstrated in a transcriptome correlation analysis. As teratoma-derived skeletal myogenic progenitors progressed from Cluster 11 to 16, they gradually shifted away from resembling Stage 2/3 cells and started to become related to, albeit remained distinct from, Stage 4/5 cells (Figure 3C). Moreover, Cluster 11 cells displayed an expression profile of stage-specific skeletal myogenic transcription factor programs [40] that largely resembled those of Stage 2/3 embryonic/fetal skeletal myogenic progenitors (Figure 3D). Taken together, these findings suggested that the formation of the skeletal myogenic lineage in ESC-derived teratomas recapitulates early skeletal myogenic development in humans.

It is notable that whereas teratoma-derived Cluster 11/12 cells might resemble Stage 2–3 embryonic/fetal skeletal myogenic progenitors, their subsequent trajectories to their respective Cluster 16 and Stage 4 (fetal week 12–18) populations appeared to diverge (Figure 3A). Cluster 16 cells, expressing skeletal myogenic specification factors, such as *MYOD1* and *MYOG* (Figure 1E), resembled a myoblast population susceptible to terminal differentiation into myotubes. In contrast, Stage 4 cells represented fetal *PAX7*^+^ progenitors leading to maturation into adult muscle stem cells (i.e., Stage 5). In fact, all *MYOD1*^+^ or *MYOG*^+^ cells across Stage 1–5 were computationally excluded from the original analysis [40]. Hence, the trajectory divergence between Cluster 11/12 to Cluster 16 and Stage 2/3 to Stage 4 might correspond to a bifurcation point of skeletal myogenic progenitor development into myofibers vs. muscle stem cells, respectively. We reasoned that the molecular changes that distinguish the trajectories into a differentiating *MYOD1*^+^ *MYOG*^+^ population (i.e., Cluster 16) from a cycling *PAX7*^+^ population (i.e., Stage 4) might include regulatory factors that promote the generation of PAX7^+^ muscle stem cells. To reveal these changes, we performed differential expression analysis between Cluster 16 teratoma-derived cells and Stage 4 fetal cells. GO analysis confirmed that Cluster 16 and Stage 4 cells were primarily separated by changes related to the skeletal myogenic differentiation process (Figure 3E). In particular, we observed in Cluster 16 cells downregulation of genes in the TGFβ (*TGFB2*) and Notch *(NOV*, *DLK1*) signaling pathways with defined regulatory roles in skeletal myogenesis [45,46,47,48,49,50] (Figure 3F). These findings suggested that regulation of TGFβ and Notch signaling pathways may be important in determining the development of ESC-derived skeletal myogenic progenitors into muscle stem cells.

### 3.4. Non-Skeletal Myogenic Populations in Human ESC-Derived Teratomas

In addition to skeletal myogenic lineage cells, we also identified in human ESC-derived teratoma populations with expression profiles resembling neuroectodermal and mesodermal cell types. Clusters 1, 2, 3, 4, 5, 7, and 10 were primarily fibroblast-like/mesenchymal and they expressed multiple extracellular matrix-related secretory factors such as *OLFML3*, *COL1A1*, and *COL5A2* (Figure S3A,B). Cluster 8 resembled chondrocytes with elevated *SOX9*, *COL2A1*, and *ACAN* expression (Figure S3C,D). The neuronal lineage was represented by Clusters 6, 9, and 14. Cluster 9 was *SOX*^+^ and Clusters 6 and 14 were enriched in early neuronal markers *DCX* and *STMN2* (Figure S3E,F). Lastly, Clusters 13 and 15 resembled neural crest derivatives: Cluster 13 being *SOX10*^+^ *S100B*^+^ *MPZ*^+^ myelinating Schwann cells (Figure S3G,H) and Cluster 15 being *TYRP1*^+^ *DCT*^+^ melanocytes (Figure S3I,J).

### 3.5. ERBB3 and CD82 Define Human Teratoma-Derived Skeletal Myogenic Progenitors

Due to the heterogeneous nature of human PSC-derived teratomas, a purification step is essential to isolate skeletal myogenic progenitors therein for downstream applications [51]. The glycoprotein CD56 is routinely used to identify endogenous muscle stem cells from human muscle biopsies [52]; however, CD56 was not skeletal myogenic-specific in human H1 ESC-derived teratomas (Figure S1E,F). From our scRNA-seq dataset, we examined previously reported putative cell surface markers for PSC-derived skeletal myogenic cells [43,52,53,54,55,56]. We found that many of these markers lacked enrichment in the skeletal myogenic populations (i.e., Clusters 11, 12, and 16), with some markers even showing increased expression in non-skeletal myogenic cells (Figure S4A).

**Figure 4:**
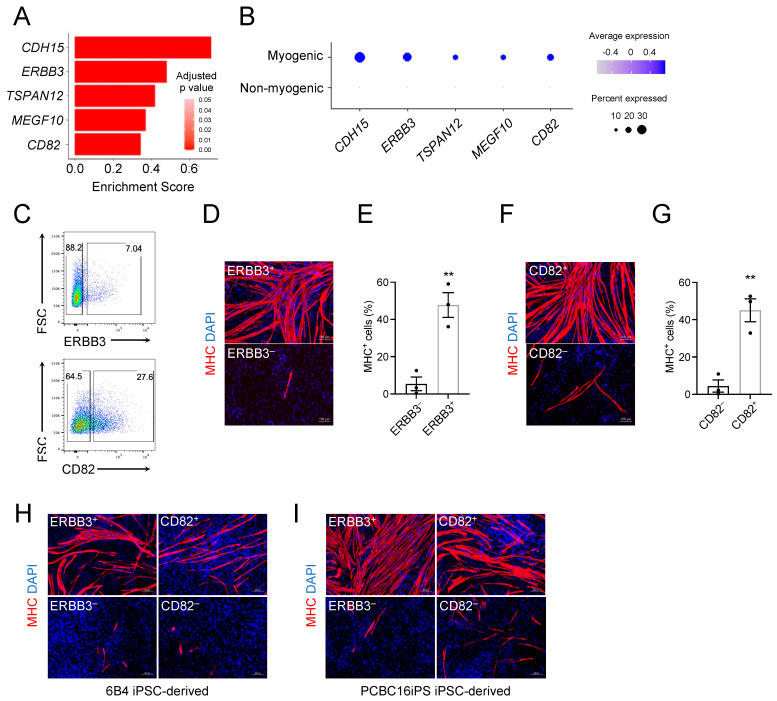
ERBB3 and CD82 are prospective surface markers for isolating skeletal myogenic progenitors from human PSC-derived teratomas. (**A**) Gene expression of candidate surface markers for skeletal myogenic cells. Expression is shown as a log-fold change in expression in skeletal myogenic clusters relative to the non-myogenic population. (**B**) Dot plot showing the enrichment of candidate surface markers in the skeletal myogenic population. (**C**) Representative FACS profiling of H1 ESC-derived teratoma cells using ERBB3 (top) and CD82 (bottom) antibodies. (**D**) Immunostaining and (**E**) quantification from three independent experiments of MHC in ERBB3^+^ and ERBB3^–^ cell fractions cultured in differentiation medium showing MHC^+^ myotubes are mostly found in the ERBB3^+^ cell fraction. Scale bar represents 100 μm. ** *p* < 0.01. Data are shown as mean ± SEM. (**F**) Immunostaining and (**G**) quantification from three independent experiments of MHC in CD82^+^ and CD82^–^ cell fractions cultured in differentiation medium showing MHC^+^ myotubes are mostly found in the CD82^+^ cell fraction. Scale bar represents 100 μm. ** *p* < 0.01. Data are shown as mean ± SEM. (**H**,**I**) ERBB3^+^ and CD82^+^ cells isolated from (**H**) 6B4 human iPSC-derived and (**I**) PCBC16iPS human iPSC-derived teratomas differentiate into MHC^+^ myotubes. Representative images from three independent teratomas are shown. Scale bar represents 100 μm.

To define the skeletal myogenic lineage in human ESC-derived teratomas for its prospective isolation, we screened the differentially expressed genes in Clusters 11, 12, and 16 that encoded surface proteins. We discovered five candidates: *CDH15*, *ERBB3*, *TSPAN12*, *MEGF10*, and *CD82* (Figure 4A,B). Among them, ERBB3 and CD82 have commercially available antibodies applicable for fluorescence activated cell sorting (FACS). We subsequently tested these antibodies in H1 ESC-derived teratoma cells, sorted both antigen-positive and negative cell fractions, and cultured them in a pro-myogenic medium (Figure 4C). We found that both ERBB3^+^ and CD82^+^ cell populations readily gave rise to MHC^+^ myotubes while ERBB3^–^ and CD82^–^ cells did not (Figure 4D–G).

To ensure that these markers were not cell line-specific, we validated these results with two additional human iPSC lines: 6B4 iPSCs (derived from adult conjunctival cells [25]) and PCBC16iPS iPSCs (derived from neonatal dermal fibroblasts [26]). We again found ERBB3^+^ and CD82^+^ cell populations enriched in MHC^+^ myotubes after cultures (Figure 4H,I). We next wished to evaluate whether combining these markers could lead to further enrichment. We found that the ERBB3^+^ CD82^+^ fraction produced the strongest enrichment in skeletal myogenesis (Figure S4B–D). Taken together, these results suggested that ERBB3 and CD82 were effective surface markers for identifying and isolating human PSC-derived skeletal myogenic progenitors produced via teratoma formation.

## 4. Discussion

Here we showed that human PSC-derived teratomas contain progenitors with potent skeletal myogenic potential. We discovered that skeletal myogenic differentiation in teratomas recapitulates human myogenesis during embryonic development. We further revealed ERBB3 and CD82 as effective surface markers for prospective isolation of teratoma-derived skeletal myogenic progenitors. The robustness and simplicity of our approach make it an attractive system to model human skeletal myogenesis.

A recent report suggested teratomas as a platform for studying multi-lineage human development [57]. Interestingly, both theirs and our current study discovered skeletal myogenic progenitors in human PSC-derived teratomas, even though teratomas were made in different locations (flank in their study and the TA muscle in ours). We decided to make teratomas in muscles because of our previous experience with mouse PSCs that teratomas formed in muscles biased toward skeletal myogenesis. Indeed, we observed multiple prominent skeletal myogenic subpopulations at different developmental stages in teratomas formed in muscles. The differences in location and timings of teratoma harvest (10-week vs. 8-week) might also explain differences in lineage composition. Other research groups also reported the presence of the skeletal myogenic lineage in teratomas formed in various locations, including testes and subcutaneous tissues, and the locations where teratomas are made might influence the efficacy of skeletal myogenic differentiation [58,59,60]. Nevertheless, the fact that skeletal myogenic progenitors were detectable in teratomas formed in different ways across multiple research groups further strengthens the robustness of the teratoma approach to derive skeletal myogenic cells from human PSCs.

Most current human PSC skeletal myogenic differentiation strategies are based on our understanding of human embryonic development, and in return, these in vitro differentiation protocols provide a useful model to study human skeletal myogenesis and muscle diseases. Interestingly, our trajectory analysis revealed that skeletal myogenic development in ESC-derived teratomas appeared to be more closely recapitulate human embryonic skeletal myogenesis than in vitro skeletal myogenic differentiation. ESC-derived teratomas might therefore provide a unique model to study human early skeletal myogenesis. It is notable that the teratoma environment is more permissive for skeletal myogenic progenitors to differentiate into myoblasts instead of mature into adult muscle stem cells. In our current study, we compared the trajectories of skeletal myogenic progenitor development towards myoblasts vs. muscle stem cells and discovered that TGFβ and Notch signaling might be important in this bifurcation process [45,46,47,48,49,50]. We believe that knowledge gained from studying skeletal myogenic development in PSC-derived teratomas will help us discover new ways to differentiate PSCs into muscle stem cells, a cell type so far elusive to produce.

Cell heterogeneity during human PSC differentiation is not unique to teratomas, as most current in vitro skeletal myogenic differentiation protocols produced many different cell types including non-skeletal myogenic [13,14,15,19]. A purification step, usually by FACS, is inevitable [51]. We found that ERBB3 and CD82 are sufficient to enrich the skeletal myogenic population in human PSC-derived teratomas. These markers might also be applicable for isolating skeletal myogenic cells in other in vitro PSC differentiation methods [20,40,44,61].

We have developed a simple and effective method of generating skeletal myogenic progenitors from human PSCs via teratoma formation. Whereas teratoma-derived cells at the current stage may not be a feasible therapeutic, by studying skeletal myogenesis in human PSC-derived teratomas, future work may uncover important developmental features that improve both in vivo and in vitro approaches of modeling human skeletal muscle development and muscular dystrophies. We envision that what we will learn from the teratoma approach will facilitate the development of future protocols for generating a clinically relevant skeletal myogenic population for treating muscular diseases without forming teratomas.

## Data Availability

The single-cell RNA-seq data generated in this study can be found in NCBI GEO under accession GSE189985.

## Supplementary Materials

The following supporting information can be downloaded at: https://www.mdpi.com/article/10.3390/cells11091589/s1, Figure S1: Skeletal myogenic development in human H1 ESC-derived teratomas; Figure S2: Single-cell RNA-seq processing; Figure S3: Heterogeneity in human teratomas; Figure S4: Establishing surface markers for isolating skeletal myogenic progenitors in human teratomas.
