# Supplementary material for: Defining the Skeletal Myogenic Lineage in Human Pluripotent Stem Cell-Derived Teratomas
Source: Cells. 2022 May 9;11(9):1589. doi: 10.3390/cells11091589 (PMC9102156; doi:10.3390/cells11091589)
Supplement: Supplementary file 1 [file cells-11-01589-s001.zip › cells-1702633-supplementary.pdf]

## Supplemental Figures

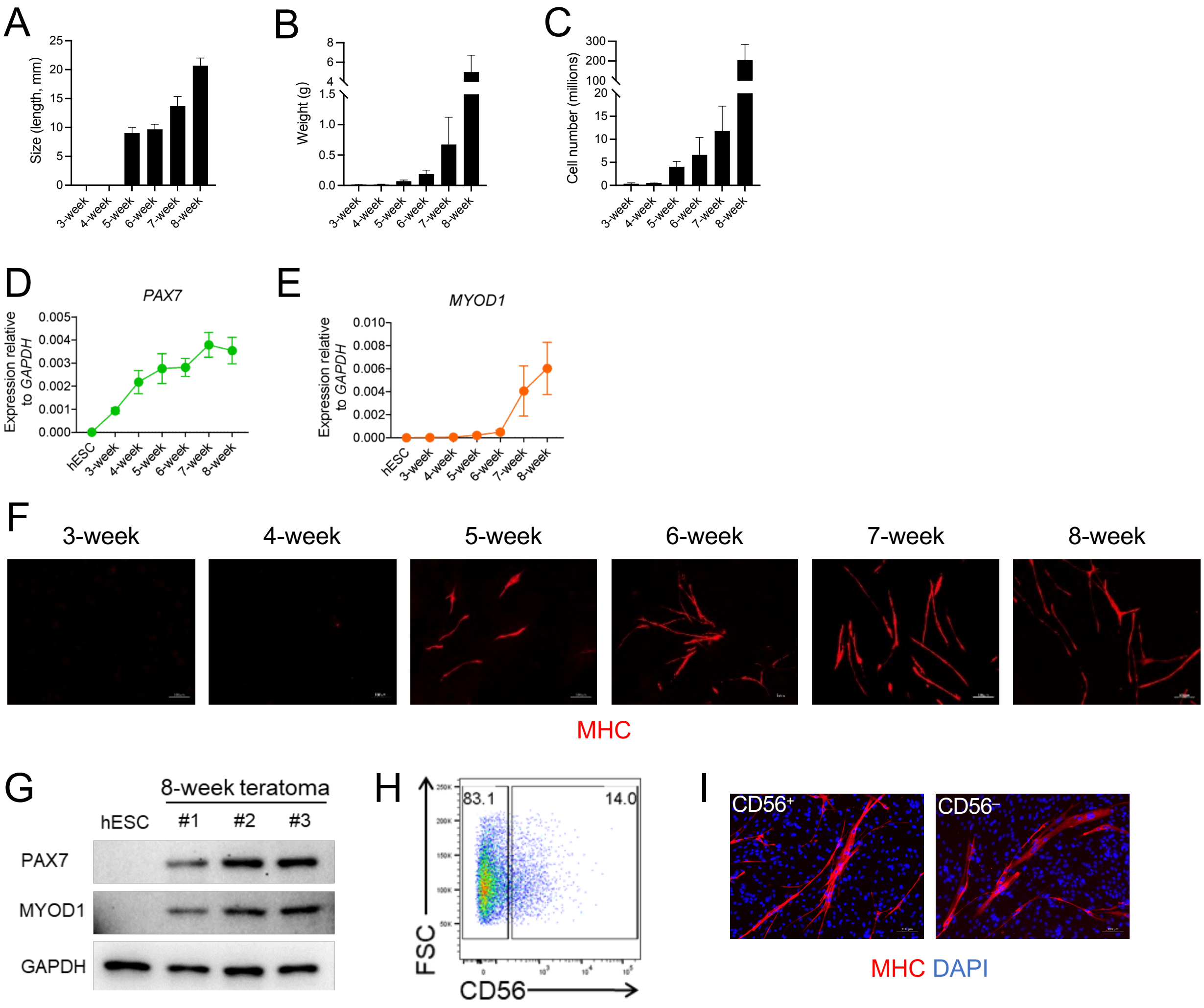

***Figure S1, related to Figure 1, Skeletal myogenic development in human H1 ESC-derived teratomas***

(A-C) Characteristics of human H1 ESC-derived teratomas: (A) size in length, (B) weight, and (C) total number of cells obtained per teratoma (n=3 independent teratomas per time point). Mean ± SEM is shown.

(D-E) Gene expression of skeletal myogenic regulatory factors (D) *PAX7* and (E) *MYOD1* in human H1 ESC-derived teratomas at various time points (n=3 independent teratomas per time point). Mean ± SEM is shown.

(F) Immunostaining of total teratoma cells in skeletal myogenic differentiation medium showing the emergence of myosin heavy chain $(MHC)^+$ myotubes starting from 5-week teratomas. Representative images from 3 independent teratomas are shown. Scale bar = 100 μm.

(G) Immunoblot showing the presence of PAX7 and MYOD1 proteins in 8-week teratomas (n=3 independent teratomas).

(H) Representative FACS plot showing CD56 expression in 8-week teratomas (from 3 independent teratomas).

(I) Immunostaining showing both $CD56^+$ and $CD56^–$ cell fractions contain $MHC^+$ skeletal myogenic cells, i.e., CD56 is unable to enrich the skeletal myogenic lineage. Representative images from 3 independent teratomas are shown. Scale bar = 100 μm.

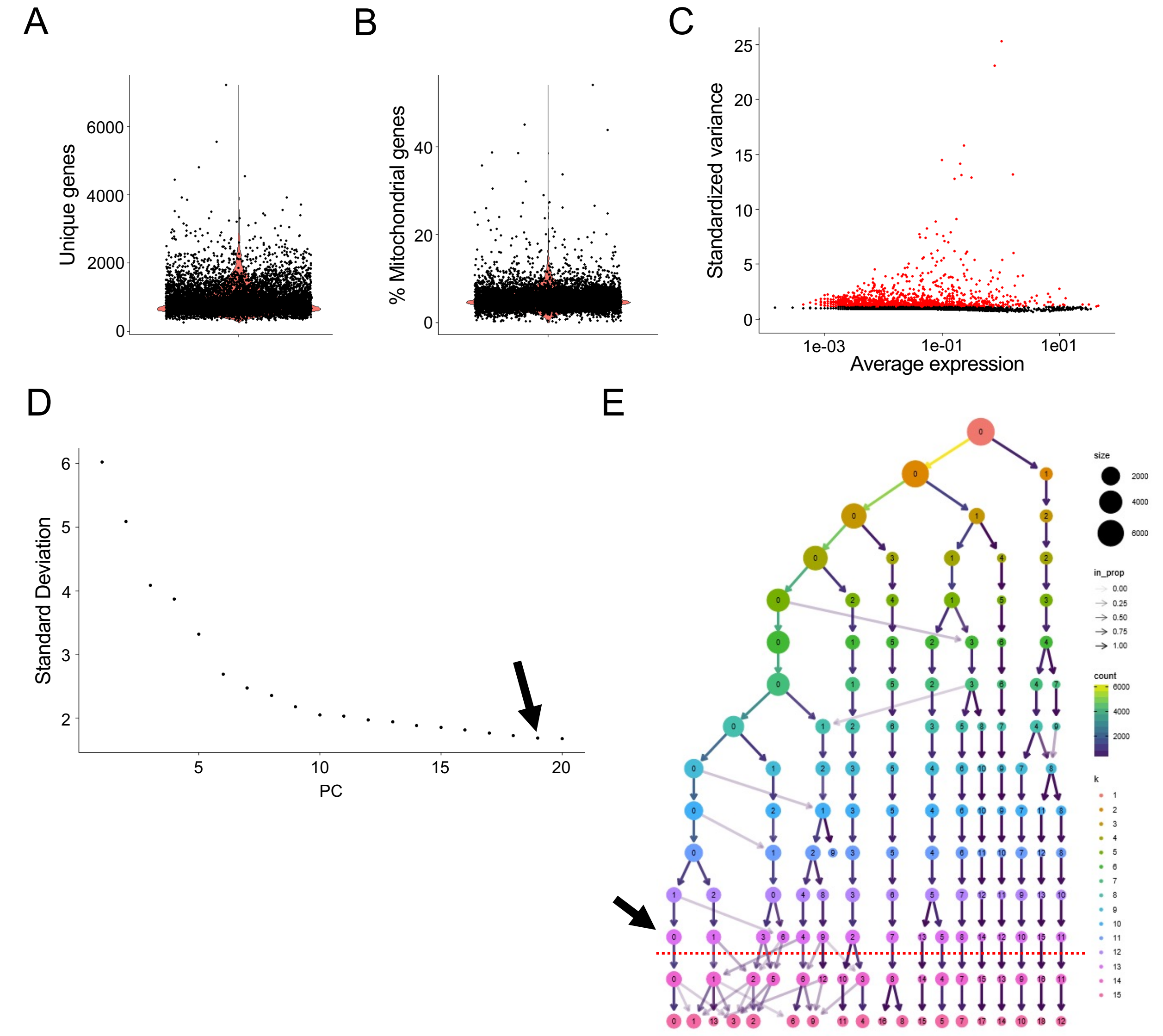


***Figure S2, related to Figure 1, Single-cell RNA-seq processing***

(A) Violin plot showing the number of unique genes detected per cell. Each dot represents a single cell.

(B) Violin plot showing the percentage of reads mapped to mitochondrial genes in each cell. Each dot represents a single cell.

(C) The 2000 most variable genes are shown based on standardized variance.

(D) Elbow plot showing the amount of deviation contained in each principal component (PC) from the 2000 most highly variable genes. Arrow indicates that 19 PCs were used in subsequent analyses.

(E) Clustree plot showing clustering hierarchy at increasing clustering resolution. Arrow indicates the utilized clustering resolution.

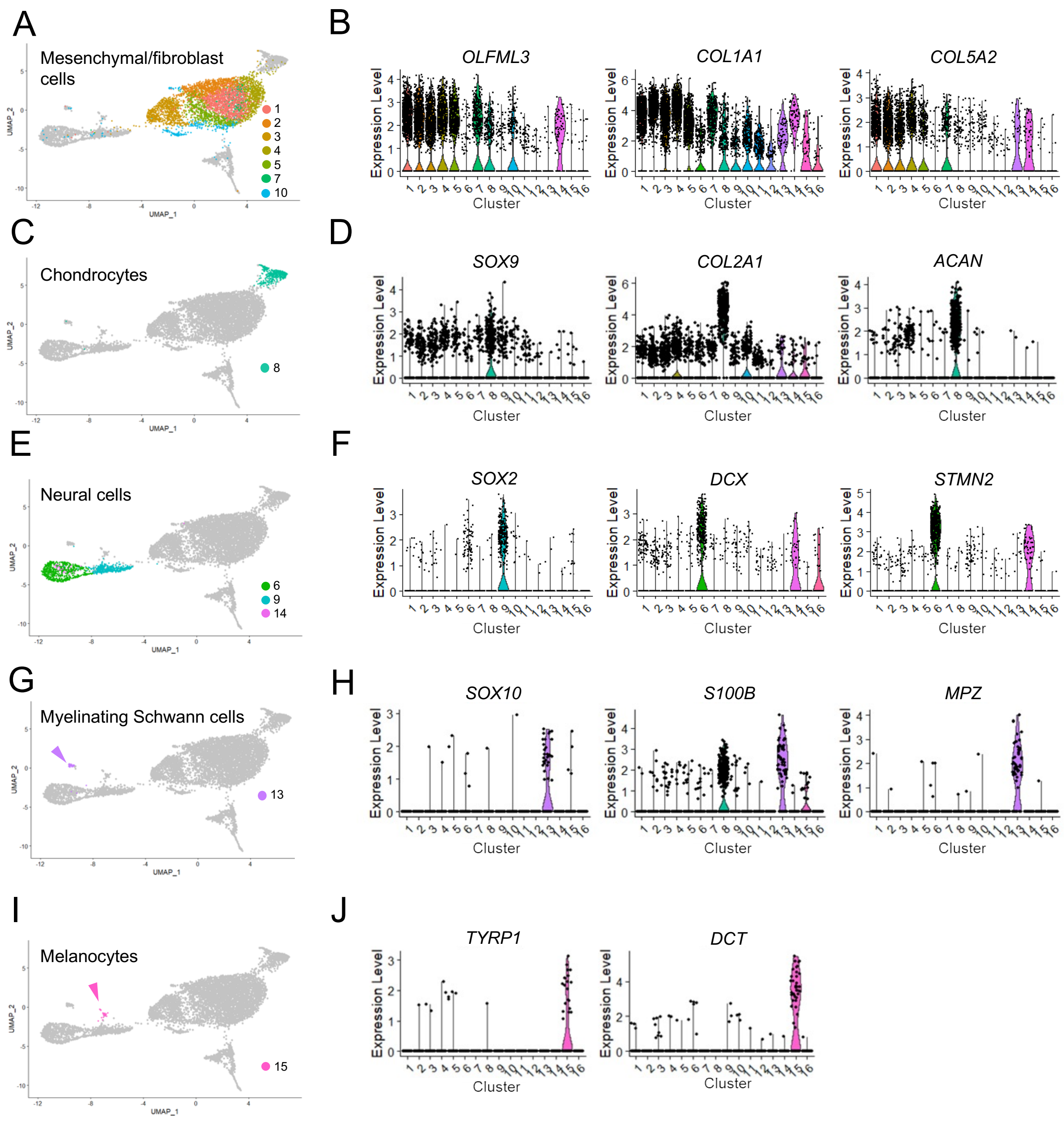


***Figure S3, related to Figure 1, Heterogeneity in human teratomas***

UMAP plots indicating clusters for (A) mesenchymal/ fibroblasts cells, (C) chondrocytes, (E) neural cells, (G) myelinating Schwann cells and (I) melanocytes, and (B, D, F, H and J) their respective violin plots of relevant genes.

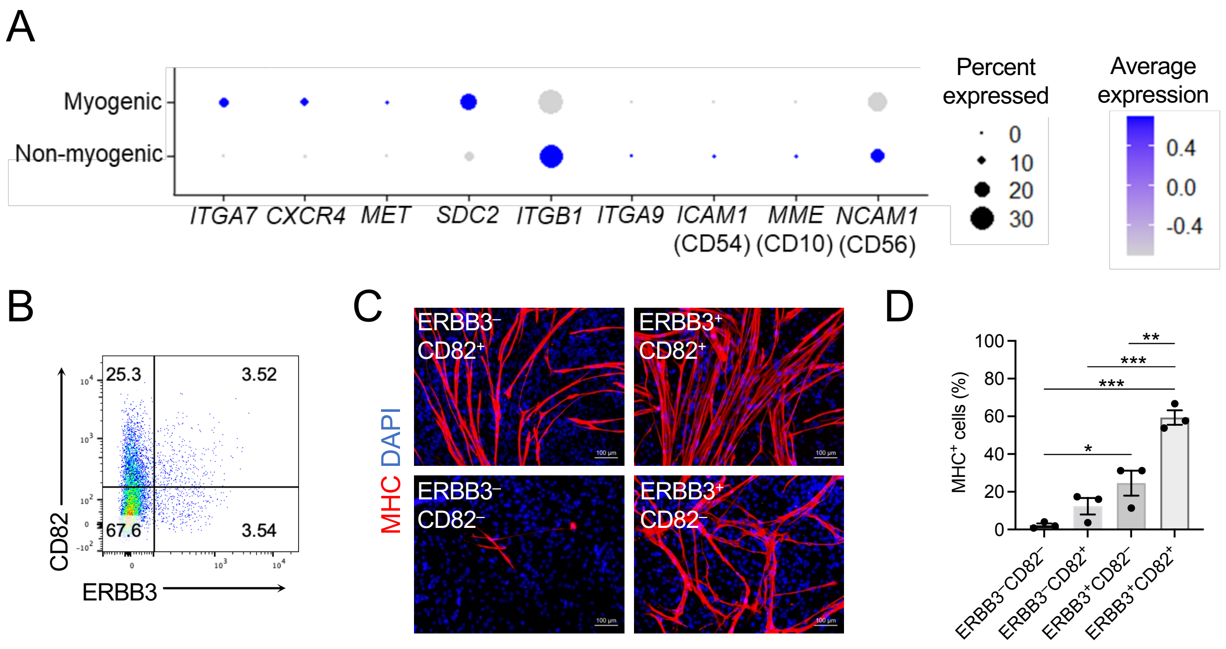


***Figure S4, related to Figure 4, Establishing surface markers for isolating skeletal myogenic progenitors in human teratomas***

(A) otplot showing gene expression profiles of previously reported potential surface markers for human H1 ESC-derived skeletal myogenic cells in myogenic and non-myogenic subsets from our single-cell RNA-seq dataset.

(B) epresentative FACS plot showing combined staining with ERBB3 and CD82 antibodies on human H1 ESC-derived teratoma cells (from 3 independent teratomas).

(C) I munostaining showing $MHC^{+}$ myotubes are mostly found in the $ERBB3^{+}$ $CD82^{+}$ cell fraction (scale bar = 100 μm), and (D) quantification of 3 independent teratomas (mean ± SEM is shown).
